# Plasmid DNA-based reverse genetics as a platform for manufacturing of bluetongue vaccine

**DOI:** 10.1128/jvi.00139-25

**Published:** 2025-03-06

**Authors:** Tendai A. M. Mlingo, Jacques Theron, Nobalanda B. Mokoena

**Affiliations:** 1 Onderstepoort Biological Products SOC Ltd., Pretoria, South Africa; 2 Department of Biochemistry, Genetics and Microbiology, University of Pretoria 56410 , Pretoria, South Africa; University of Michigan Medical School, Ann Arbor, Michigan, USA

**Keywords:** bluetongue virus, orbivirus, reverse genetics, vaccine, chimeric, immunogenicity

## Abstract

Control of bluetongue disease is predominantly through vaccination with licensed inactivated or live-attenuated vaccines (LAVs). Manufacturing of LAVs in endemic countries requires formulation with a high number of serotypes for effective protection. Herein, we evaluated a plasmid DNA-based reverse genetics platform for manufacturing a multivalent vaccine. The synthetic vaccine was characterized by a common BTV1 backbone, with exchange of outer capsid proteins. Recombinant South African vaccine serotypes 1, 5, and 14 were rescued by exchanging the VP2 protein on the backbone. BTV6 rescue was achieved following the exchange of VP2 and VP5 proteins. The particle sizes were comparable to commercial vaccines of respective serotypes. BTV1, BTV5, and BTV6 had distinct growth profiles compared to commercial vaccines, while BTV14 was indistinguishable. Stability and shelf-life determination under various storage conditions showed that commercial vaccines were more stable. Formulated antigens were evaluated for vaccine safety and immunogenicity in sheep. Serotyped BTV1 monovalent vaccine was safe, as no clinical signs were observed. Neutralizing antibodies (nAbs) were induced on day 14 and peaked at 32 on day 28. The multivalent synthetic vaccine containing four serotypes elicited BTV6 nAbs from day 21 with a titer of 52, which decreased to 33 by day 42. BTV1 elicited a weak immune response with a titer of 1 on day 42. No nAbs were detected against BTV5 and BTV14. This is a first report comparing reverse genetics-derived antigens with commercial vaccines. Data generated on production yields, stability, and immunogenicity demonstrated that some serotypes can be implemented as novel synthetic vaccines using this platform.

**IMPORTANCE:**

Vaccination is the most effective control strategy for viral diseases that affect livestock. To date, only live-attenuated and inactivated vaccines have been licensed for control of bluetongue (BT). This study demonstrated the use of reverse genetics as a possible platform for BTV vaccine production. Data generated in the study contribute toward the advancement of an alternative manufacturing platform for licensing of BT vaccines. Information on production yields and stability of synthetic vaccines in comparison to the conventional products demonstrated that optimization is required for some serotypes to fully translate the reverse genetics platform for manufacturing the BTV vaccine. The study highlighted the safety and immunogenicity of vaccines manufactured using the plasmid DNA-based reverse-genetics platform.

## INTRODUCTION

Bluetongue virus (BTV) is the prototype member of the *Orbivirus* genus in the family *Sedoreoviridae* (1). The arthropod-transmitted virus causes a non-contagious hemorrhagic disease in wild and domestic ruminants (2, 3). Bluetongue is classified as a notifiable disease by the World Organisation for Animal Health (WOAH) due to its social and economic impact in agriculture. There are currently 36 typical, atypical, and putative BTV serotypes (4, 5) based on serotype-specific neutralization of the viral outer-capsid protein (6, 7). The icosahedral virus consists of three protein layers: the outer capsid layer (VP2 and VP5), the core–surface layer (VP7), and the sub-core shell (VP3) (8).

Strategies to control BTV include annual vaccination with licensed inactivated or live-attenuated vaccines (LAV) (9). A model vaccine against BT disease should be cost-effective, safe, efficacious against multiple serotypes, and elicit long-lasting immunity (10, 11). The LAVs have been extensively used for the prophylactic immunization of animals against bluetongue disease and elicit a long-lasting protection against specific serotypes after a single dose. Cross-protection has been demonstrated across specific serotypes when using LAVs (12). However, challenges, such as viremia, reversion to virulence, and reassortment with field strains, persist (13). Inactivated vaccines are often considered safer options, as no clinical manifestations in target animals are reported. However, a short-lived immune response and ineffective processes to inactivate viruses remain as drawbacks (13, 14). To address these deficiencies, reverse genetics platforms were developed for the generation of novel vaccines. Subunit vaccines, such as VP2, have been extensively evaluated in different expression hosts, such as bacteria (15, 16), baculoviruses (17, 18), and yeast (19). Additionally, insect- and plant-produced virus-like particles have been applied for vaccine manufacturing (20–22). Genetically modified viruses, such as modified vaccinia Ankara, canarypox, myxomavirus, capripoxvirus, and adenovirus, have also been assessed as gene delivery systems for viral-vectored vaccines (23–27). An RNA-based reverse genetics platform for *in vitro* synthesis of RNA transcripts from complementary DNA (cDNA) clones has been evaluated for the development of live or inactivated synthetic serotyped antigens rescued by exchanging the outer capsid proteins VP2 and VP5 on the backbone of a different serotype (28–30). This platform was applied for the development of Entry Competent Replication Abortive (ECRA) vaccines. A mutation in the essential segment 9 gene rendered the virus replication incompetent but immunogenic and protective in sheep (10, 31, 32). Furthermore, Disabled Infectious Single Animal (DISA) vaccines with NS3/NS3a knockout mutations were safe and efficacious when evaluated in sheep and cattle (33–35). However, production costs are escalated by low titers and an essential complementary cell line (11). To minimize challenges presented by RNA-based platforms, a PCR-based reverse genetics approach was successfully evaluated for the recovery of recombinant viruses. In the two-step transfection approach, initial transfection with helper plasmids for the expression of proteins required for virus assembly and replication was followed by transfection with BTV PCR amplicons (36). An entirely plasmid DNA-based reverse genetics platform for vaccine development was successfully applied for the rescue of infectious serotyped vaccines. In this approach, mammalian cells constitutively expressing T7 RNA polymerase were transfected with 10 plasmid constructs representative of an entire BTV genome (37).

In this study, the plasmid DNA-based reverse genetics platform was evaluated for application in the manufacturing of a synthetic vaccine against bluetongue disease. Owing to the large number of serotypes in endemic areas, the production process is labor-intensive due to a requirement to propagate serotypes individually and the variable yields obtained with individual viruses. The South African BTV1 reference strain was selected as the backbone serotype for the candidate vaccine. Reverse genetics vaccine serotypes 1, 5, 6, and 14 were rescued successfully by exchanging the capsid proteins. The growth kinetics of most synthetic viruses were distinct from the parent strains. The commercial vaccine serotypes were demonstrated to have higher yields and more stable than the synthetic viruses. The synthetic BTV1 monovalent vaccine proved to be safe and elicited neutralizing antibodies (nAbs) on day 21. The multivalent vaccine formulated with four serotypes elicited nAbs against serotype 6. Serotype 1 induced a weak immune response, while serotypes 5 and 14 nAbs were not detected.

## MATERIALS AND METHODS

### Cells and viruses

Baby hamster kidney cells (BHK-21 clone 13) and African green monkey kidney (Vero) cells (European Collection of Authenticated Cell Culture, United Kingdom) were grown in Glasgow’s Minimum Essential Medium (GMEM) (Gibco) supplemented with 5 or 10% (v/v) adult bovine serum (CellSera), 2.5 µg/mL amphotericin B (Oxoid), and 0.1 mg/mL streptomycin (Merck). Cells were maintained at 37°C with 5% CO_2_. The BSR cells (a clone of BHK-21) stably expressing bacteriophage T7 RNA polymerase (38) were maintained in MEM (Gibco) supplemented with 2 mM L-glutamine (Thermo Fisher Scientific), 0.1 mg/mL streptomycin, 2.5 µg/mL amphotericin B, and 5% (v/v) adult bovine serum. Every second passage on BSR-T7 cells was supplemented with 1 mg/mL of Geneticin (Invitrogen). BSR-T7 cells were used for the recovery of BTV particles. Synthetic viruses were amplified on BHK-21 or Vero cells.

BTV1 was selected as the genetic backbone for the recovery of virus particles (37). Virus vaccine stocks of BTV serotypes 1, 3, 4, 5, 6, 7, 12, and 14 (Table 1) manufactured by Onderstepoort Biological Products (OBP), Pretoria, South Africa were used. BTV antigens were propagated in BHK-21 cells in serum-free GMEM supplemented with antibiotics.

**TABLE 1:** Genome accession numbers of selected serotypes for synthetic vaccine

BTV1 reference strain		Serotypes for candidate vaccines							
		BTV1	BTV3	BTV4	BTV5	BTV6	BTV7	BTV12	BTV14
Seg-1	FJ969719								
Seg-2	FJ969720	MZ130556	MZ395173	MZ130566	MZ215862	MZ130576	MZ215872	MZ130586	MZ130596
Seg-3	FJ969721								
Seg-4	FJ969722								
Seg-5	FJ969723								
Seg-6	FJ969724	MZ130560	MZ395177	MZ130570	MZ215866	MZ130580	MZ215876	MZ130590	MZ130600
Seg-7	FJ969725								
Seg-8	FJ969726								
Seg-9	FJ969727								
Seg-10	FJ969728								

### cDNA synthesis

Total RNA was extracted using Tri Reagent (LS) according to the manufacturer’s instructions. cDNA was synthesized using the protocol described by Potgieter et al. (2009). Single-stranded RNA was precipitated from total RNA in a final concentration of 2 M lithium chloride at 4°C for 16 h, then centrifuged at 16,000 × *g* for 30 min at 4°C. The supernatant was collected in a new tube, and dsRNA was purified using the MinElute Gel Extraction Kit (Qiagen). Purified dsRNA was ligated to the “anchor primer” (300 ng), PC3-T7 (Table 2) (39), using T4 RNA ligase (TaKaRa) as described by the manufacturer. MinElute gel extraction columns were used to purify samples from unbound oligonucleotides according to the manufacturer’s instructions. A final volume of 10 µL purified PC3-T7 ligated dsRNA was collected. Denaturation of ligated dsRNA was carried out in a final concentration of 30 mM mercury hydroxide and kept at 37°C for 30 min. The reaction was terminated with 1% (v/v) β-mercaptoethanol. First-strand cDNA synthesis was performed at 42°C for 45 min, and the synthesis was terminated at 55°C for 15 min. The reverse transcription reaction contained 15 U AMV-RT, 20 U RNAse inhibitor, 1.4 mM dNTPs, and 1× cDNA buffer (50 mM Tris–HCl pH 8.3, 10 mM MgCl_2_, 70 mM KCl, and 30 mM β-mercaptoethanol) in a final volume of 30 µL. When the synthesis of the first strand was completed, untranscribed RNA was eliminated with the addition of sodium hydroxide to a final concentration of 0.1 M and incubated at 65°C for 30 min. A final concentration of 0.1 M Tris–HCl pH 7.5 was added, followed by HCl (Sigma-Aldrich) to a final concentration of 0.1 M. Annealing of the cDNA was completed at 65°C for up to 3 h. PCR amplification was performed in a reaction containing 1× TAQ buffer (TaKaRa), 2.5 U TAQ DNA polymerase (TaKaRa Bio), 0.2 mM dNTPs, 25 µM PC2 primer (Table 2), and 5 µL cDNA. The reaction was maintained at 72°C for 10 min to allow complete synthesis of ends before denaturation was conducted at 94°C for 2 min. PCR amplification consisted of 35 cycles with denaturation at 94°C for 30 s, annealing at 65°C for 30 s, extension at 72°C for 4 min, and a final extension at 72°C for 5 min. The amplified product was resolved on a 1% (w/v) agarose gel stained with ethidium bromide in 1× TAE buffer. Amplification of full-length BTV genome segments was completed using serotype-specific primer pairs (Table 2) and Phusion high-fidelity DNA polymerase (Thermo Fisher Scientific) as recommended by the manufacturer.

**TABLE 2:** Oligonucleotides for polymerase chain reaction amplification

Oligonucleotide	Sequence^*a*^ 5′−3′	Restriction enzyme	GC content (%)	Annealing temp (ºC)
BTV1VP2F	AGACAcgtctcgTATAGTTAAAATAGTAGCGCGATGGATG	BsmBI	42.5	60
BTV1VP2R	AGACAcgtctcaGGCCGTAAGTCTAATAGTGCGCG		54.3	
BTV4VP2F	AGACAcgtctcgTATAGTTAAAAGAGTGTCCCACAATGGA		42.5	61
BTV4VP2R	AGACAcgtctcaGGCCGTAAGTGTAAGAGGCCACA		54.3	
BTV6&14VP2F	AGACAcgtctcgTATAGTTAAATTAGTTTCGTGATGGATG			58
BTV6&14VP2R	AGACAcgtctcaGGCCGTAAGTTGATTAGTTCGTG			
BTV12VP2F	AGACAcgtctcgTATAGTTAAAAGTTGCGAGGATGGAGGA		45.0	61
BTV12VP2R	AGACAcgtctcagGCCGTAAGTTGAAGCCG		56.7	
BTV7VP5F	AGACAcgtctcgTATAGTTAAAAAGTGTCCTCATCATCGC		42.5	60
BTV7VP5R	AGACAcgtctcaGGCCGTAAGTGTAAGCGC		56.7	
BTV3VP5F	AGACAcgtctcgTATAGTTAAAAAGWTCCCYATGATCGCG			60
BTV3VP5R	AGACAcgtctcaGGCCGTAAGTRKAAGTCC			
BTV5VP2F	AGACAgaagacagTATAGTTAAAAGCTTCTCAGGATG	BbsI	37.8	55
BTV5VP2R	AGACAgaagacagGGCCGTAAGTGTAWGCT		51.7	
BTV5VP7R	AGACAgaagacagGGCCGTAAGTGTAATCC		50.0	
BTV5VP5F	AGACAgaagacagTATAGTTAAAAAGWTCCCYATGATCGCG		40.2	58
BTV5VP5R	AGACAgaagacagGGCCGTAAGTRKAAGTCC		51.6	
BTV3VP2F	AGACAgaagacagTATAGTTAAAAACGCTGTCCCGAG		43.2	58
BTV3VP2R	AGACAgaagacagGGCCGTAAGTGWAAACGTG		50.0	
BTV7VP2F	AGACAgaagacagTATAGTTAAAAAGGACCTCGCCA		42.9	58
BTV7VP2 R	AGACAgaagacagGGCCGTAAGTTTAAGGCC		51.6	
BTV4VP5F	AGACAgaagacagTATAGTTAAAAAGTRTTCTCCTACTCGC		37.8	57
BTV4VP5R	AGACAgaagacagGGCCGTAAGTGTAAGCTTC		50.0	
BTV6 &14VP5F	AGACAgaagacagTATAGTTAAAAAGWTCCCYATGATCGCG		40.2	58
BTV6&14VP5 R	AGACAgaagacagGGCCGTAAGTRKAAGTCC		51.6	
BTV1VP5F	AGACAacctgcagtcTATAGTTAAAAAGTGCACCCTTAG	BspMI	41.0	59
BTV1VP5R	AGACAacctgcagtaGGCCGTAAGTGTAAGTGC		51.5	
BTV12VP5F	AGACAacctgcagtcTATAGTTAAAAAGCAGCCACC		44.4	59
BTV12VP5 R	AGACAacctgcagtaGGCCGTAAGTGTAAGTAG		50.0	
PC3-T7	GGATCCCGGGAATTCGGTAATACGACTCACTATATTTTTATAGTGAGTCGTATTA			
PC2	CCGAATTCCCGGGATCC			
BTV Seg1 forward primer	TTAAAATGCAATGGTCGCAAT C			
BTV Seg1 reverse primer	TCCGGATCAAGTTCACTCC			
BTV Seg1 probe	FAM-CCGTGCAAGGTGC-MGB			

^*a*^ Restriction sites are shown in lowercase.

### Synthetic BTV genome segments

The cDNA for BTV segment 2 from all serotypes, as well as segment 6 of BTV12, contained *Bsm*BI, *Bb*SI, or *Bsp*MI restriction sites. These BTV segments were synthesized by Bio Basic (Canada). The sequence of the genome segments was codon-optimized for expression in Chinese hamster ovary cells, and *Bsm*BI restriction sites flanked the full-length segments at the 5′ and 3′ termini. All other *Bsm*BI cut sites were silenced to facilitate cloning of genome segments into pUC57.

### Generation of recombinant rescue plasmids

Synthetic BTV genome segments, RT-PCR amplicons, and the pRG15 rescue vector (37) were digested with restriction enzymes *Bsm*BI, *Bb*SI, or *Bsp*MI to generate compatible ends and enable cloning of the genome segments under control of the T7 RNA polymerase promoter of pRG15. Individual genome segments were ligated into pRG15 using T4 DNA ligase (Thermo Fisher Scientific) at 4°C according to the manufacturer’s instructions. Recombinant plasmids were designated pRG15_BTV1VP2_ according to the serotype and full-length genome segment cloned. Sanger sequencing of the recombinant plasmids to confirm the BTV insert was performed by Inqaba Biotechnical Industries (PVT, Ltd.).

### Rescue of synthetic virus particles

The BSR-T7 cell monolayers were seeded in T25 flasks or six-well plates to reach 50–80% confluency in 24 h at 37°C with 5% CO_2_. Transfection was conducted using Lipofectamine LTX Reagent (Invitrogen). Briefly, plasmids with BTV genome segments (500 ng) and Lipofectamine LTX (15 µL) were each diluted separately in 150 µL of OptiMEM serum-free media, then incubated at room temperature for 5 min. The constructs and reagent were combined, and 14 µL PLUS reagent was added. Following 45 min of incubation at room temperature, the transfection mixture was added to monolayers of BSR-T7 cells, and the mixture made up to 3 mL using OptiMEM. Serotyped antigens comprised eight or nine BTV1 backbone genome segments together with outer capsid-encoding genome segments (VP2 only or VP2 and VP5). The DNA plasmids of selected serotypes were co-transfected, and cell cultures examined daily for cytopathic effects (CPE). Synthetic viruses recovered were designated “B1” according to the backbone serotype, followed by the serotype of exchanged VP2 and VP5. For instance, _B1_BTV5VP2 depicts a reassortant BTV1 backbone with BTV5 segment 2.

### Viral quantification of rescued antigens

Rescued antigens were quantified using the modified tissue culture infective method of Spearman–Kärber (40) and an adapted viral plaque titration assay (41).

#### Spearman and Kärber methods

Briefly, 10-fold serial dilutions of BTV (10^−1^ to 10^−8^) were prepared in serum-free GMEM supplemented with antibiotics, and 100 µL of each dilution was added to 96-well plates in six replicates. Vero cells were prepared in suspension in GMEM media with antibiotics and 10% (v/v) adult bovine serum and 100 µL was added to the antigens. The plates were incubated at 37°C with 5% CO_2_ for 5 days. The viral titer was calculated according to the Spearman and Kärber methods (40).

#### Viral plaque quantification

Briefly, 10-fold serial dilutions of rescued synthetic BTV (10^−1^ to 10^−6^) were prepared in serum-free GMEM supplemented with antibiotics. Vero cells were seeded in six-well plates to reach 100% confluency in 24 h. Media were discarded from the cultures, and 1 mL of each viral dilution was added in the plates in duplicate and incubated at 37°C with 5% CO_2_ for 1 h. Following viral adsorption, 4 mL of 1% (w/v) agarose (Laboratorios Conda) overlay was added to each well, and plates were incubated at 37°C for 5 days prior to staining with 2 mL of 1% (v/v) neutral red dye (Sigma Aldrich) prepared in 0.5% (w/v) agarose. Plaques were tallied, and the viral titer was calculated.

To determine the plaque diameter, nine to 42 plaques were measured using ImageJ software (https://imagej.net/ij/). To plaque purify antigens, well-separated BTV plaques were picked from the agar overlay using Pasteur pipettes, and the agar plug was transferred to microfuge tubes containing 0.5 mL GMEM. To facilitate viral release, agar plugs were vortexed and stored at 4°C overnight. Plaque-purified recombinant viruses were amplified on Vero cells, and viral stocks were stored at 4°C until required.

### Virus morphology

Transmission electron microscopy (TEM) was used to view virus particles. Synthetic and vaccine antigen culture supernatants were centrifuged at 900 × *g* for 15 min, and the supernatant was collected in a sterile 1.5 mL centrifuge tube. Samples were then centrifuged at 16,000 × *g* for 45 min, and the supernatant was discarded. Pellets were rinsed and resuspended in double-distilled water, then negatively stained with an equal volume of 3% (v/v) phosphotungstic acid for 15 s before mounting on Formvar carbon-coated grids. Excess stain on the grids was gently blotted away with Whatman paper. Viral particles were viewed with a JEOL–JEM-1400Flash transmission electron microscope operated at 100 kV.

### Virus typing

Typing of the synthetic antigens was performed using virus neutralization assays (42) and nucleotide sequencing of segment 2 (29). VP2 is the major determinant of serotype and neutralization owing to the location of the majority of epitopes (43). Virus neutralization tests were carried out in 96-well microtiter plates using an adapted method from Sailleau and colleagues (42). Briefly, two-fold serially diluted synthetic antigens were titrated against a constant amount of type-specific serum previously heat-inactivated at 56°C for 30 min. After incubation of the mixture at 37°C for 1 h, Vero cells in cell media supplemented with serum and antibiotics were added. The cultures were incubated at 37°C with 5% CO_2_ for up to 7 days. The reciprocal of the highest viral dilution that gave 50% protection was determined to be the viral titer. For nucleotide sequencing, genomic RNA was extracted from recovered viral progeny using Tri reagent. RT-PCR amplification of BTV segment two was performed in a 50 µL reaction containing 2 µL SuperScript III RT/Platinum Taq Mix (Invitrogen), 1× reaction buffer, ≤1 µg RNA, and 0.4 µM of the appropriate serotype-specific segment 2 forward and reverse primers (Table 2). Reverse transcription was performed at 55°C for 30 min. Following pre-denaturation at 94°C for 2 min, a PCR amplification was carried out in 40 cycles with denaturation at 94°C for 15 s, annealing at the appropriate temperature (Table 2) for 30 s and extension at 68°C for 4 min. A final extension at 68°C for 5 min completed the reaction. Sanger sequencing of the gel-purified amplicons to confirm the serotype was performed by Inqaba Biotechnical Industries (PVT, Ltd.).

### Virus growth kinetics

Virus growth kinetics of the rescued antigens in comparison with viruses of the same serotype in the vaccine were evaluated using the real-time cell analysis (RTCA) system (xCELLigence, software 2.0.0.1301; ACEA Biosciences) (44). Vero cells (100 µL) at a concentration of 10,000 to 50,000 cells/mL were seeded into each well of the 16-well *E-*plates. An additional 100 µL of GMEM with 10% (v/v) serum was added, and plates were loaded onto the system. Cells were cultured at 37°C with 5% CO_2_ for 48 h, and cell index (CI) values measured every 60 min. Vero cell monolayers with a cell index value of 8 and above were infected with 100 µL of individual viruses at a titer of 1.00E + 04 TCID_50_/mL and incubated at 37°C with 5% CO_2_. After 1 h of incubation, 100 µL of GMEM without serum was added, and the cells were further incubated for an additional 150 h or until the cell death phase was reached in all samples. All samples were analyzed in duplicate, and three independent experiments were conducted. The CI_50_ value indicating 50% of the maximum CI for each antigen analyzed was determined. Parallel cell monitoring was conducted in 96-well plates. To compare growth kinetics of the synthetic and vaccine strains using virus yields, Vero cells were seeded in 96-well plates and cultured at 37°C with 5% CO_2_ for 48 h. The confluent monolayers were inoculated with BTV antigen at a titer of 1.00E + 04 to 5.00E +04 TCID_50_/mL in triplicate in a final volume of 200 µL of GMEM. Samples were collected every 24 h for 3 days, and titers (TCID_50_) were determined using a modified Spearman–Kärber formula. The growth kinetics of the commercial and synthetic virus particles were compared.

### Virus stability assessment

The stability of synthetic viruses in comparison to the respective serotype antigens used in the production of registered vaccines was assessed at 4 (cold-chain temperatures), −40 (cryopreservation temperature), and −80°C (storage temperature). Samples evaluated included the following: harvested antigen, antigen formulated with a buffered stabilizer, and antigen formulated with a buffered stabilizer and lyophilized. The integrity of viral particles was assessed using TEM weekly for 4 weeks. To determine the shelf life, synthetic and commercial vaccine antigens kept at the various conditions were quantified weekly for at least 10 weeks using the Spearman and Kärber methods as described above. The shelf life of the synthetic and vaccine products was evaluated by comparing the titers.

### Safety in sheep

Thirty healthy Merino sheep aged 4 to 5 months were sourced from Langfontein farm (Mpumalanga, South Africa). Pre-screening of sheep was conducted before delivery using a BTV VP7 group-specific competitive enzyme-linked immunosorbent assay (cELISA) (45) in a WOAH reference virology laboratory at the Agricultural Research Council—Onderstepoort Veterinary Institute (ARC-OVI). BTV naïve and healthy sheep were transported to the vector-controlled Onderstepoort Veterinary Animal Research Unit for the safety and immunogenicity study. The sheep were acclimatized in the facility for 8 days, and then randomly assigned to six treatment groups with five animals each. Two vaccine strategies were applied where sheep were vaccinated with a monovalent or multivalent vaccine (Table 3). Monovalent preparations consisted of the synthetic _B1_BTV1VP2 vaccine and the licensed OBP BTV1 monovalent vaccine. To evaluate the replication potential and capacity of synthetic antigens to induce clinical signs, the BTV1 South African reference strain was incorporated in the study. The synthetic and licensed OBP multivalent vaccines were composed of serotypes 1, 5, 6, and 14. The lyophilized vaccines were reconstituted to a final dose titer of 1.00E + 04 PFU/dose and administered subcutaneously on the inner right thigh using a 21-gauge needle. The equivalent volume of vaccine diluent was administered in the naïve control group. Animals were examined daily for reactions at the site of injection and clinical signs (Table S1). Rectal temperatures were monitored twice daily for 14 days following vaccination, then once daily for the duration of the study. A temperature reading of ≥40°C on two consecutive days was interpreted as a temperature reaction. Scores were assigned daily according to the temperature and clinical signs observed on the nose, mouth, and hooves (Table S1). All temperature recordings of ≥40°C were awarded one point for each degree of 40°C and above.

**TABLE 3:** Synthetic and licensed vaccines used as monovalent or multivalent formulations

	Group	Sheep
Monovalent	_B1_BTV1VP2	T3
		T4
		T6
		T13
		T107
	Synthetic BTV1 reference strain	T32
		T45
		T48
		T50
		T60
	OBP BTV1	T37
		T57
		T67
		T83
		T91
Multivalent	Synthetic serotyped multivalent BTV vaccine^*a*^	T63
		T85
		T99
		T104
		T106
	OBP multivalent BTV vaccine^*a*^	T11
		T19
		T24
		T41
		T46
	Placebo	T29
		T72
		T117
		T128
		T129

^*a*^ Contains serotypes 1, 5, 6, and 14.

To assess viremia, blood samples were collected in EDTA tubes (4 mL) on days 0, 3, 5, 7, 10, and 14 and stored at 4°C before analysis. BTV RNA was confirmed using real-time reverse transcriptase quantitative PCR (RT-qPCR) targeting segment 1. Total RNA was extracted from EDTA blood samples using the 5× MagMax Pathogen RNA/DNA Kit (Thermo Fisher Scientific) on the KingFisher Flex magnetic particle processor (Thermo Fisher Scientific) according to the manufacturer’s instructions. The 342 bp product was amplified using the VetMAX-Plus One-step RT-PCR Kit (Thermo Fisher Scientific) with previously described primers and probes (Table 2) (46). The 25 µL reaction contained 0.4 µM BTV Seg1 forward primer, 0.4 µM BTV Seg1 reverse primer, 0.12 µM BTV Seg1 probe, 2× RT-PCR buffer (50% v/v), 25× RT-PCR enzyme mix (4% v/v), and 5 µL BTV RNA. Reverse transcriptase was carried out on Quant Studio 5 (Thermo Fisher Scientific) at 48°C for 10 min, followed by denaturation at 95°C for 10 min. Amplification consisted of 40 cycles at 95°C for 15 s and 60°C for 45 s. A positive reaction was defined by a cycle threshold (Ct) value below 35.

### Immunogenicity

BTV immunity conferred following vaccination with the monovalent and multivalent vaccines was evaluated (Table 3). Blood samples were collected in serum separator tubes (10 mL) on days 0, 3, 5, 7, 10, 14, 21, 28, 35, and 42 and assessed for BTV antibodies against serotypes 1, 5, 6, and 14. Sera from the coagulated blood samples were collected by centrifugation at 3000 × *g* for 30 min. The serum samples were transferred to sterile 2 mL cryotubes and heat-inactivated in a 56°C water bath for 30 min. The samples were stored at −20°C prior to serological assessment.

### Serological analysis

#### Evaluation of BTV antibodies using cELISA

Group-specific antibodies were estimated using a commercially available BTV cELISA kit (VMRD, USA). Detection of VP7 antibodies was carried out at the ARC-OVI as described by the manufacturer. The absorbance readings were established at 620, 630, or 650 nm. The inhibition percentage of the samples was calculated as follows: Inhibition % = [1 − (Sample OD ÷ Negative control OD)] × 100. Samples with inhibition values of 60% and above were interpreted as positive for VP7 antibodies.

#### Evaluation of BTV antibodies using the serum neutralization test

Serotype-specific and cross-neutralization antibody titers were determined using an adapted serum neutralization test in microtiter plates (45). Briefly, two-fold 50 µL dilutions were performed in triplicate in serum-free GMEM supplemented with antibiotics. An equal amount of partially attenuated BTV field antigen available at OBP was added to each well to a final concentration of 100 TCID_50_. To allow neutralization, samples were kept at 37°C with 5% CO_2_ for 1 h. Thereafter, 100 µL of Vero cells at a concentration of 400,000 cells/mL was added, and plates were kept at 37°C with 5% CO_2_. Plates were monitored daily for cytopathic effects for up to 5 days. The reciprocal of the highest serum dilution that gave 50% protection against the virus was determined as the antibody titer.

## RESULTS

### Recovery of recombinant viruses

This study aimed to rescue synthetic serotyped BTV particles with a common backbone derived from the BTV1 South African reference strain. For this purpose, recombinant reverse genetics vectors representing the entire genome of the South African reference strain BTV1 (37) were adopted as the reverse-genetics backbone vectors. Recombinant pRG15 reverse-genetics vectors containing synthetic codon-optimized or cDNA copies of genome segments 2 and 6 of selected BTV serotypes were constructed (Fig. S1), and the integrity of the constructs was verified by sequence analysis. The deduced amino acid sequence of the respective cloned genome segments was identical to the parental genome, with the exception of segment 6 of serotype 7 and segment 2 of serotype 14. Single non-synonymous mutations were identified in BTV7 VP5, where isoleucine was substituted by threonine at residue position 320, and in BTV14 VP2, where glycine was substituted by arginine at amino acid position 395.

**Fig 1:**
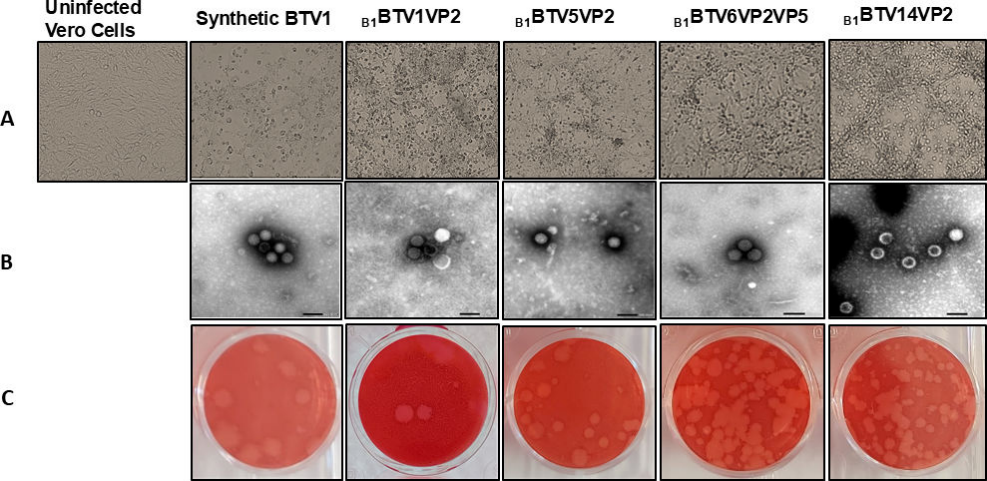
Characterization of synthetic viruses. (**A**) Cytopathic effects of rescued BTV on Vero cells imaged between 24 and 72 h post-infection. Uninfected Vero cells were included as a control. (**B**) Electron micrographs of synthetic reassortant viral particles viewed showing fully formed BTV particles (bar, 100 nm). (**C**) The plaque morphology of recovered BTV progeny was assessed on infected Vero cells overlaid with agarose and imaged after 5 days.

To recover synthetic serotyped viruses, BSR cells constitutively expressing T7 RNA polymerase were transfected with reverse genetics vectors representative of the entire BTV genome. The synthetic serotype 1 reference strain was successfully rescued, and chimeric viruses were created by exchanging antigenic VP2 segment alone or a combination of VP2 and VP5 segments on the BTV1 backbone. The following serotyped viruses were successfully recovered from vaccine strains after exchange of the VP2 segment only: _B1_BTV1VP2, _B1_BTV5VP2, and _B1_BTV14VP2. Serotyped BTV6 (_B1_BTV6VP2VP5) recombinant vaccine was rescued by exchanging both VP2 and VP5 capsid proteins (Fig. 1A through C). Microscopic examination of transfected BSR cells showed CPE after 3–5 days. Rescued antigens were then amplified on Vero cells, and CPE was observed 1–3 days after virus infection (Fig. 1A). Fully assembled viruses, as well as particles at various stages of assembly, were observed using TEM (Fig. 1B). BTV serotypes 3, 4, 7, and 12 could not be rescued by exchanging VP2 only or both VP2 and VP5.

### Virus morphology

The morphology of BTV synthetic particles was compared to OBP vaccine antigens (Fig. 1B). Synthetic serotype 1 virus particles _B1_BTV1VP2 and the vaccine strain were similar in size, ranging from 54 to 78 nm (Table 4). Likewise, synthetic BTV14 particles ranging from 52 to 68 nm were congruent with BTV14 vaccine particles that ranged between 51 and 72 nm (Table 4). Synthetic BTV5 particles were 53 to 72 nm in diameter, while the vaccine strain ranged from 46 to 73 nm. Serotype 6 rescued antigens ranging from 53 to 69 nm, and its respective vaccine strain was 58 to 75 nm in diameter. Fully formed synthetic viruses, as well as particles at various stages of assembly or disassembly, were observed.

**TABLE 4:** BTV particle size and plaque size of lyophilized antigens

Antigen	Particle size range (nm)	Plaque size (mm)	
		Mean	Range
BTV1 reference strain	54–69	1.55	0.48–3.87
_B1_BTV1VP2	55–78	1.19	0.29–3.47
_B1_BTV5VP2	53–72	1.75	0.59–3.09
_B1_BTV6VP2VP5	53–69	1.44	0.55–2.36
_B1_BTV14VP2	52–68	1.51	0.57–2.42
BTV1 vaccine	54–68	1.93	0.98–3.31
BTV5 vaccine	46–73	3.86	2.02–5.07
BTV6 vaccine	58–75	2.25	0.58–3.12
BTV14 vaccine	51–72	1.59	0.58–2.75

Additionally, viral plaque assays were carried out on Vero cells, and the sizes of the plaques formed by vaccine and synthetic antigens were measured and compared (Fig. 1C; Table 4). Synthetic reference and reassortant serotype 1 antigens produced plaques with a mean diameter of 1.55 and 1.19 mm, respectively. The mean diameters of serotype 5 and 6 plaques were determined at 1.75 and 1.44 mm, respectively, while synthetic BTV14 plaque diameters averaged at 1.51 mm. Notably, plaques formed by registered vaccines had larger mean diameters: BTV1 vaccine, 1.93 mm; BTV5 vaccine, 3.86 mm; and BTV6 vaccine, 2.25 mm. BTV14 vaccine had a smaller average diameter of 1.59 mm. The data sets from the vaccine and synthetic antigens alluded to differences during viral replication.

### Typing of antigens

Serotype confirmation of synthetic antigens was carried out using virus neutralization tests with type-specific antisera. Titers of 80 and 40 were obtained for _B1_BTV5VP2 and _B1_BTV14VP2, respectively. Typing of the synthetic reference strain, _B1_BTV1VP2, and _B1_BTV6VP2VP5 yielded high titers at ≥640. A comparison of the partial sequence data of segment 2 RT-PCR products obtained from synthetic viruses with their respective parent viruses confirmed the serotypes of the antigens. In summary, all serotypes were successfully confirmed with molecular and serological techniques.

### Growth kinetics

**Fig 2:**
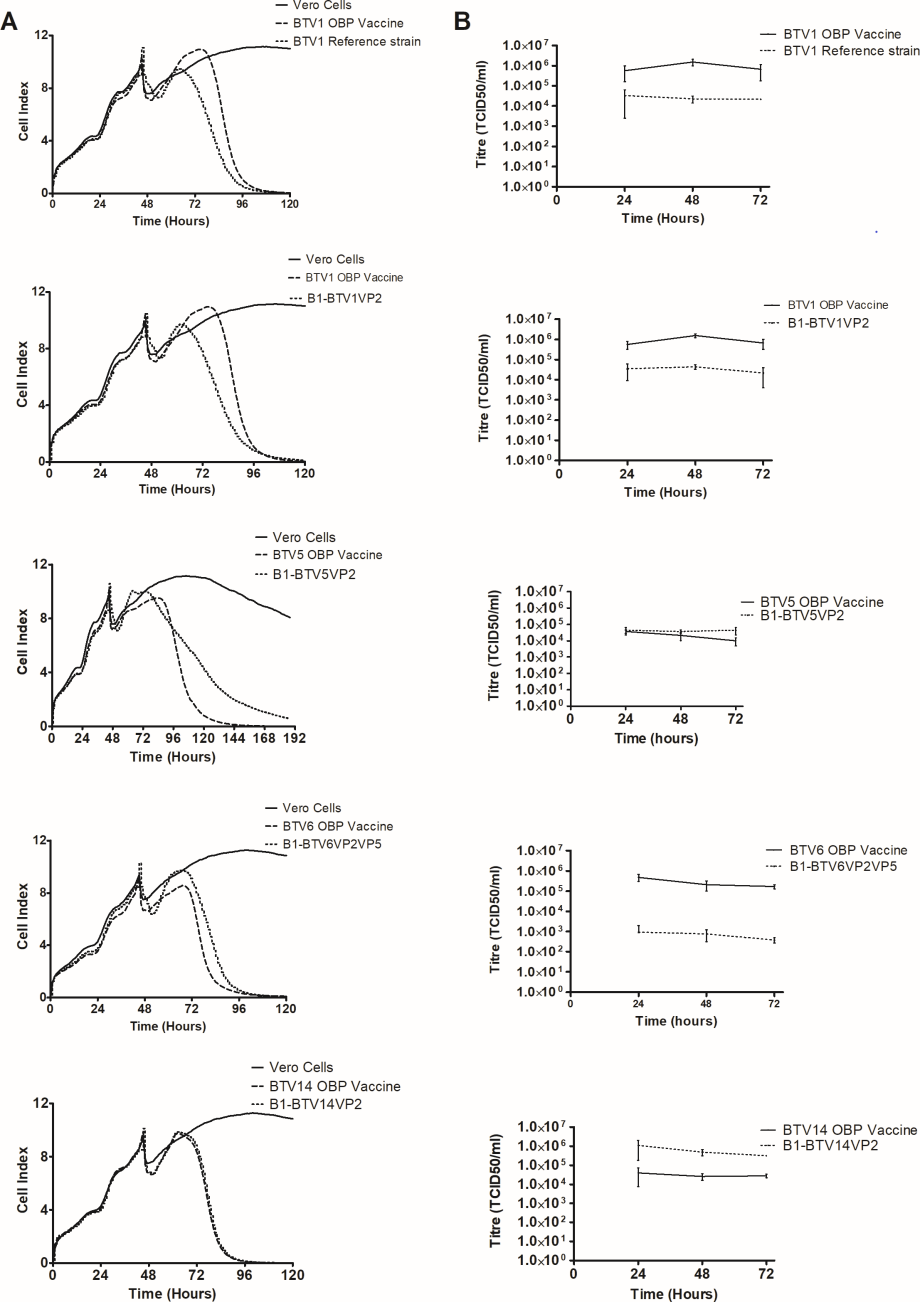
Growth profiles of synthetic and licensed vaccine antigens. (**A**) Monitoring of cytopathic effect using the real-time cell analysis (xCELLigence) of Vero cells as a function of the cell index (CI) over time for 96 h. An increase in CI depicted growth and attachment of Vero cells, and a decrease in CI indicated cell death and, therefore, detachment of cells from the surface. (**B**) Antigen yields were monitored in 96-well plates. Virus titers were determined every 24 h.

The RTCA system uses electrical impedance technology to monitor cell adhesion, viability, growth, and morphology (47, 48). To compare replication kinetics of licensed vaccine strains with the synthetic viruses, cells were cultured in *E*-plates and inoculated with antigen in the exponential growth phase. Assays were conducted in duplicate, and average CI values were calculated for all the wells. A reduction in the CI value depicted cell death due to viral replication. The CI_50_ value indicates 50% of the maximum CI for each antigen analyzed. Control Vero cells were not infected with BTV and grew to a maximum CI of 11.28 (Fig. 2A). An analysis of the synthetic vaccine serotype 1 antigens showed comparable replication kinetics to the synthetic BTV1 reference strain. At approximately 35 h post-infection, the CI_50_ values for the _B1_BTV1VP2 and BTV1 reference strains were 4.59 and 4.73, respectively. In comparison, the BTV1 vaccine strain had a CI_50_ value of 5.48 after approximately 41 h. This implied a slower growth rate by the vaccine strain. The CI_50_ values of the BTV5 vaccine (55 h post-infection) and the synthetic strain (51 h post-infection) were 5.16 and 7.13, respectively. This indicated a more rapid replication by the vaccine strain. A comparison of the CI values at 120 h post-infection showed that the vaccine strain (0.79) had almost reached 100% CPE, while the synthetic virus (4.21) was still replicating. The CI_50_ value of the BTV6 vaccine strain was 4.27 approximately 31 h after infection. In comparison, the synthetic antigen had a CI_50_ value of 4.85 approximately 36 h post-infection. This indicated a more rapid replication rate by the vaccine strain. In contrast, the vaccine and synthetic BTV14 replication kinetics were indistinguishable. At approximately 32 h post-infection, the CI_50_ values of the vaccine and synthetic strains were 4.88 and 4.93, respectively. In summary, synthetic serotypes 1, 5, and 6 exhibited distinct growth profiles from their respective registered vaccine.

Virus yields during replication were evaluated in Vero cells seeded in 96-well plates. The titers were determined every 24 h for up to 72 h, and the performance of synthetic and vaccine strains was analyzed (Fig. 2B). A comparison of the yields of the vaccine types was performed at 72 h post-infection for all serotypes. Vaccine strains for serotypes 1 and 6 had titers of 6.58E + 05 and 1.70E + 05 TCID_50_/mL, respectively. The BTV1 synthetic reference strain and _B1_BTV1VP2 each had lower titers of 2.14E + 04 TCID_50_/mL, while synthetic BTV6 had a low yield of 3.78E + 02 TCID_50_/mL. Serotype 5 vaccine and synthetic strains had yields of 9.78E + 03 and 4.38E + 04 TCID_50_/mL, respectively. Serotype 14 vaccine antigen had a titer of 2.60E + 04 TCID_50_/mL, and the corresponding synthetic vaccine had a titer of 4.87E + 05 TCID_50_/mL. The observations demonstrated the varying performance of commercial vaccine strains in comparison to synthetic strains. The licensed vaccine strains gave higher yields for serotypes 1 and 6, while serotype 14 and serotype 5 yields were lower.

### Stability of synthetic viruses

#### Shelf life of liquid products

**Fig 3:**
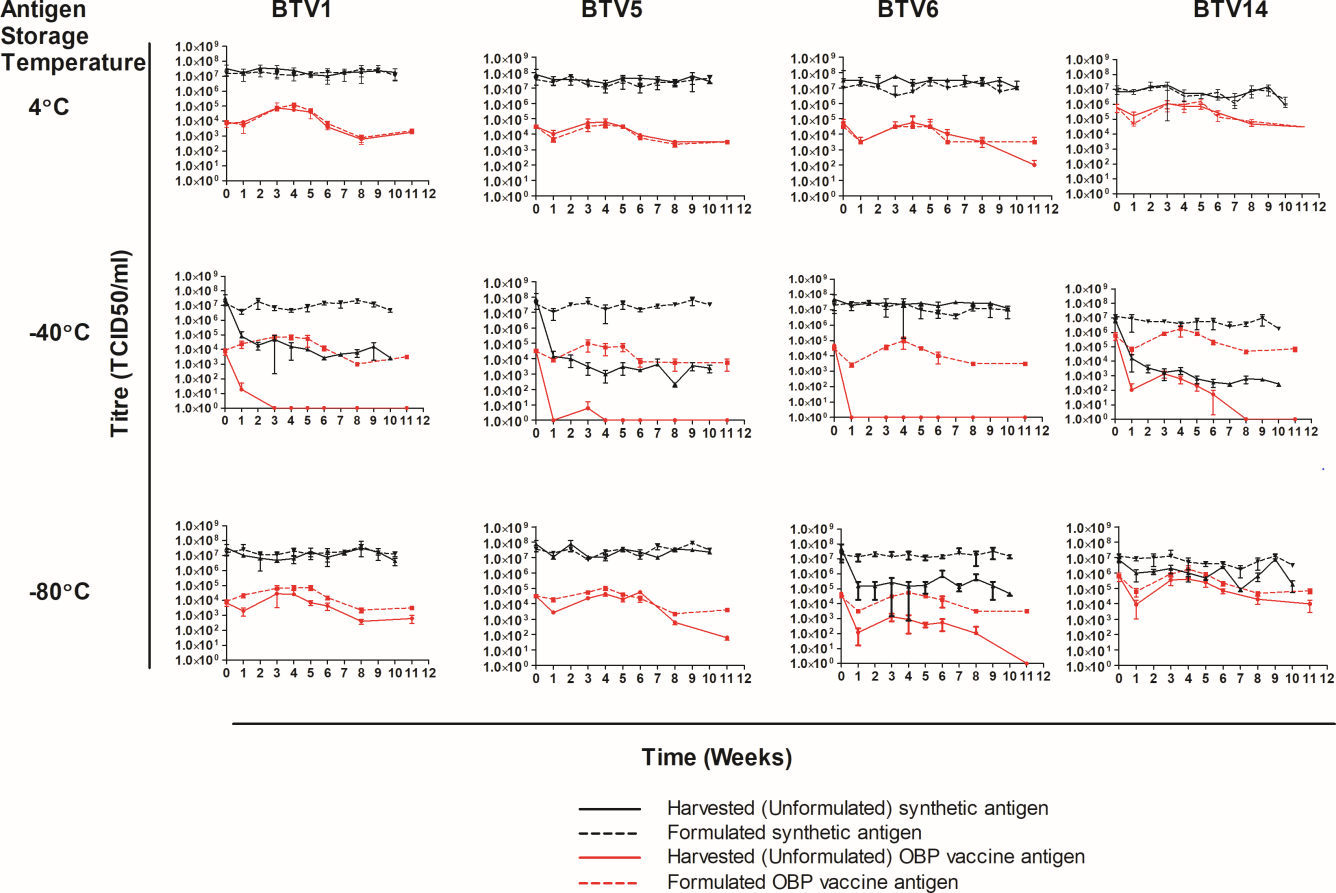
Stability assessment of licensed vaccine and synthetic BTV antigens (liquid formulation). Antigens formulated with stabilizer and unformulated were kept at 4, −40, and −80°C. Antigen stability was determined weekly using the Spearman and Kärber methods.

The BTV vaccine shelf-life recommendation for liquid and freeze-dried antigen by the WOAH is 1 and 2 years, respectively (45). The antigen integrity and shelf life of synthetic and vaccine antigens were comparatively assessed at three temperatures under three different storage conditions. To investigate synthetic and vaccine antigen stability, samples were stored with and without a stabilizer and lyophilized at 4, −40, and −80°C. The samples were evaluated weekly by viral quantification over a period of 10 weeks. The formulated and unformulated licensed vaccine strains were stable at 4°C (Fig. 3). In comparison, titers of synthetic viruses were maintained up to 4 weeks, and a slight decrease was observed from week 5. TEM analysis indicated a greater proportion of disassembled synthetic particles (Fig. S2) in comparison to the licensed vaccine. At −40°C, vaccine and synthetic strains were more stable when formulated with a stabilizer. Unformulated synthetic antigen could not be detected after 3 weeks of storage. An exception was _B1_BTV14VP2, which was detectable up to week 8 owing to the high titer at week 0. Unformulated vaccine antigens for serotypes 1, 5, and 14 experienced a marked decrease in titer after 1 week of storage. At −80°C, formulated synthetic and vaccine strains maintained higher titers in comparison to unformulated antigens. Although vaccine strains for three serotypes (1, 5, and 14) were relatively stable across the 10 weeks, a steady decrease in titer was observed in synthetic antigens. In conclusion, vaccine antigens were more stable and had longer shelf lives relative to synthetic antigens. It was evident that synthetic antigens rescued at higher titers had extended shelf lives.

#### Stability of lyophilized antigens

**Fig 4:**
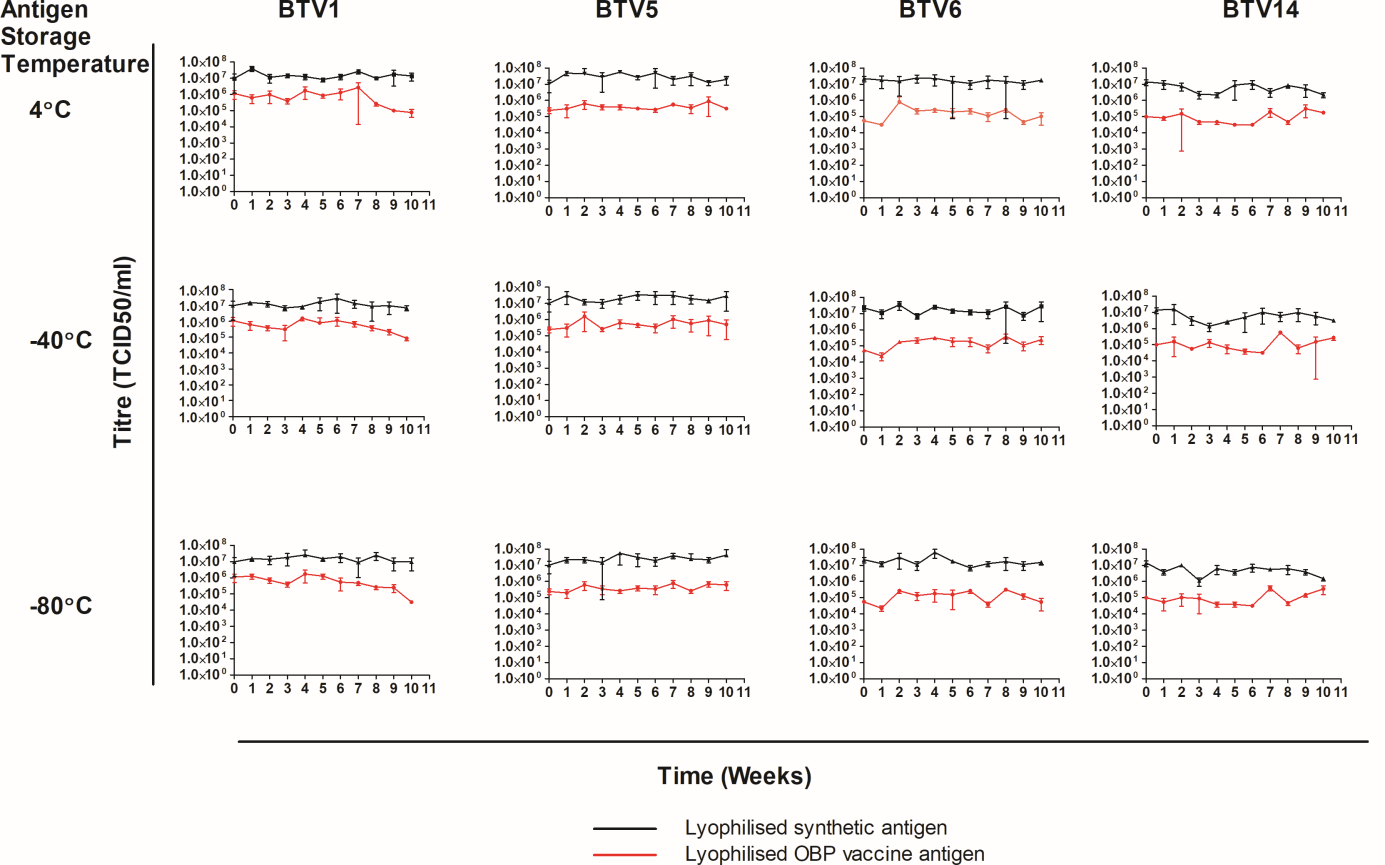
Stability assessment of lyophilized licensed vaccine and synthetic BTV antigens. The Spearman and Kärber methods were applied weekly to assess the stability of the antigens kept at 4, −40, and ­80°C.

Synthetic antigens were formulated with a stabilizer and lyophilized using the same protocol applied to licensed vaccines. The lyophilized synthetic antigens were monitored at three temperatures (Fig. 4) with the aim of identifying the optimum storage temperature. Although licensed vaccine antigens had higher titers at week 0, comparable stability profiles for synthetic and vaccine antigens were evident across all three temperatures (4, −40, and −80°C). An exception to this was synthetic BTV1, where, in comparison to the three synthetic viruses, a decrease in titer was observed between weeks 4 and 10 at the three temperatures analyzed.

#### Vaccine safety in sheep

**Fig 5:**
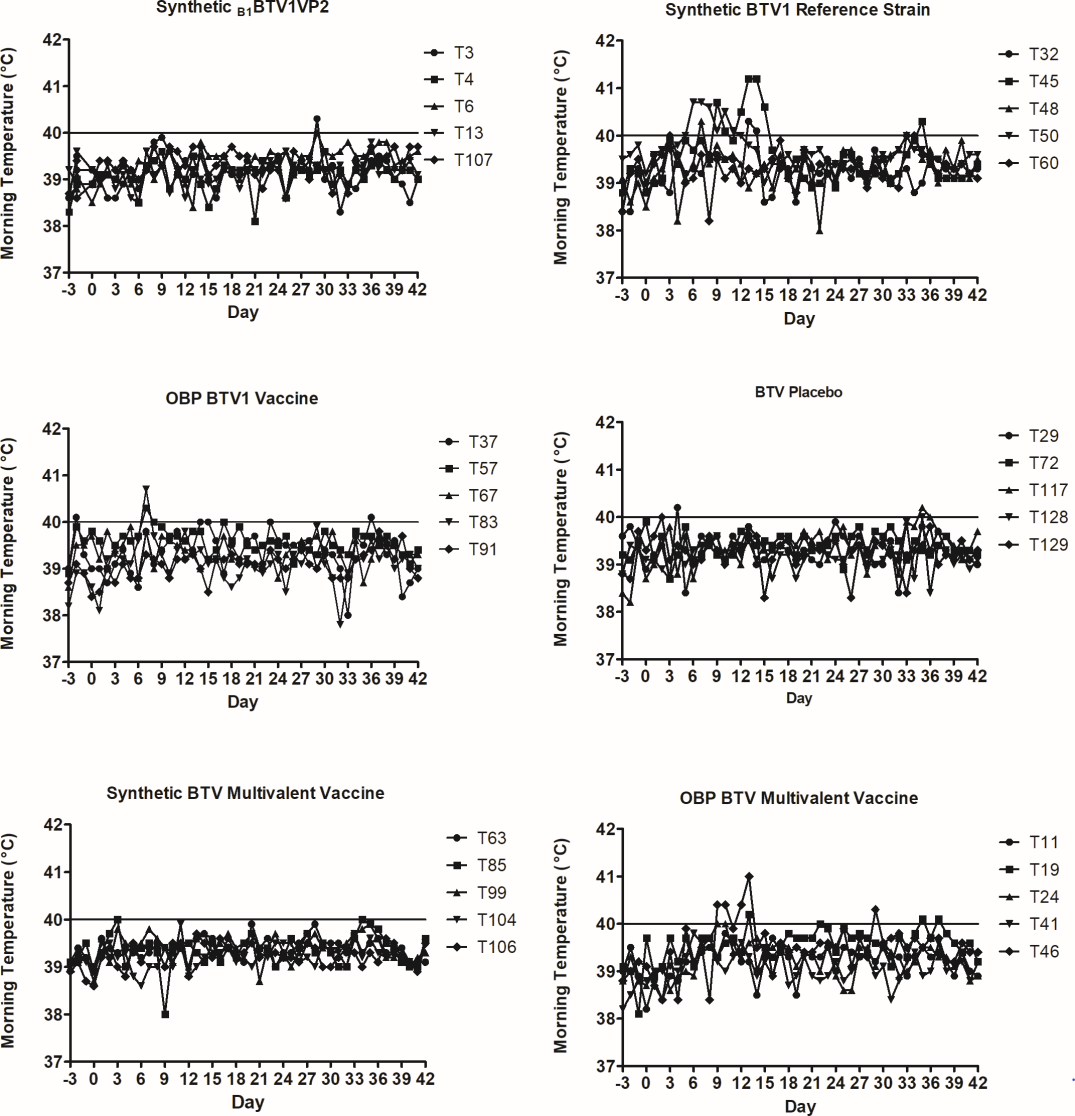
Morning temperatures in vaccinated sheep. Sheep were vaccinated with synthetic or licensed BTV1 monovalent vaccines, and the temperature was monitored daily for 42 days.

The safety of the synthetic monovalent (serotype 1) or multivalent (serotypes 1, 5, 6, and 14) vaccines was evaluated in sheep. Positive control groups consisted of the licensed monovalent and multivalent vaccines with corresponding serotypes. The synthetic monovalent and multivalent vaccines did not induce temperature reactions in sheep (Fig. 5). When compared to the corresponding monovalent licensed vaccines, temperature reactions were observed in sheep T37 (days 14 to 15) and T57 (days 7 to 8). Similarly, two sheep (T24 and T46) in the multivalent licensed vaccine group had temperature reactions between days 9 and 13. In comparison, three sheep (T32, T45, and T50) inoculated with the reference strain gave temperature reactions between days 5 and 15. No BT-related temperature reactions were evident in the placebo group.

With the exception of one sheep, clinical signs were not observed in sheep vaccinated with synthetic monovalent and multivalent vaccines. Sheep T6 vaccinated with the monovalent serotyped BTV1 vaccine presented with a transient swollen lip and mouth and was prostrated throughout day 17. In the corresponding monovalent positive control group, one sheep (T37) presented with slight hyperemia on the lower jaw for 24 h on day 5. Furthermore, transient nasal discharges were observed in sheep vaccinated with the licensed multivalent vaccine or reference strain on days 14 (T46) and 17 (T45), respectively. No BT-related clinical signs were observed in the placebo group. A calculation of the average clinical score for each treatment group for the first 21 days following vaccination (Fig. S3) gave low scores below 1.

#### BTV RNA detection using RT-qPCR

**Fig 6:**
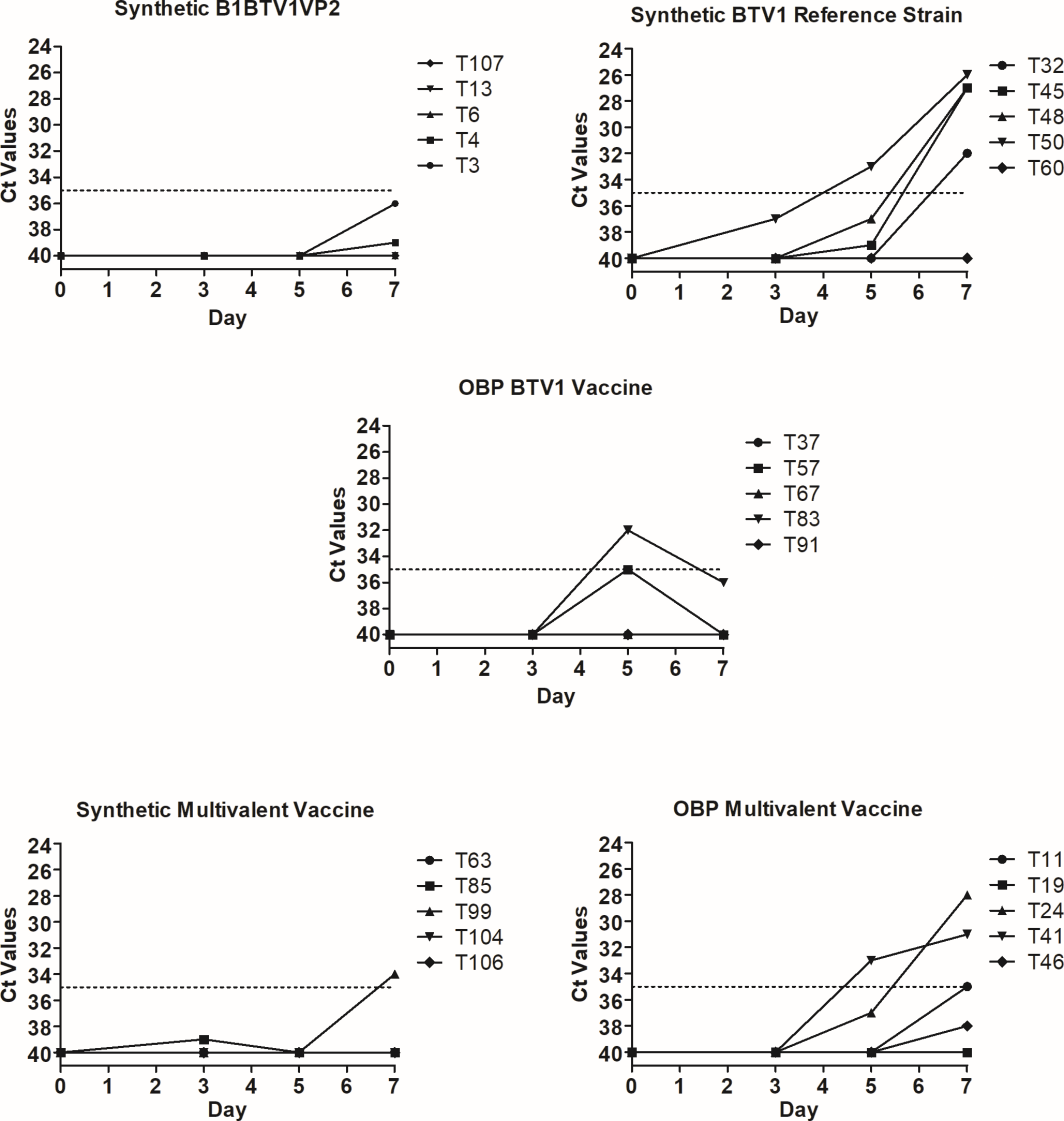
Real-time RT-qPCR cycle threshold values. Sheep were vaccinated subcutaneously with synthetic or licensed vaccines. Blood samples collected on days 0, 3, 5, and 7 days post-vaccination were evaluated for viremia by RT-qPCR. The cycle threshold (Ct) values are indicated.

The detection of BTV segment 1 RNA using RT-qPCR was used to assess viremia following vaccination. Samples with cycle threshold (Ct) values below 35 were defined as positive for BTV RNA. An assessment of RNA 7 days post-vaccination demonstrated the absence of viremia in sheep vaccinated with _B1_BTV1VP2 (Fig. 6). In comparison, one sheep in the monovalent control group had a high Ct value of 32 on day 5 that declined on day 7. An assessment of the multivalent vaccine groups showed that a single sheep vaccinated with the synthetic vaccine had a high positive Ct value of 34 on day 7 as compared to two sheep vaccinated with the licensed multivalent vaccine with Ct values of 28 and 31. In comparison, four sheep inoculated with the reference strain were viremic after 7 days and had Ct values that ranged between 26 and 32. This was attributed to the synthetic virus’ partial attenuation. In summary, analysis of the rectal temperature, clinical signs, and viremia in vaccinated animals established the synthetic monovalent and multivalent vaccines as safe for use in sheep.

#### Vaccine immunogenicity

**Fig 7:**
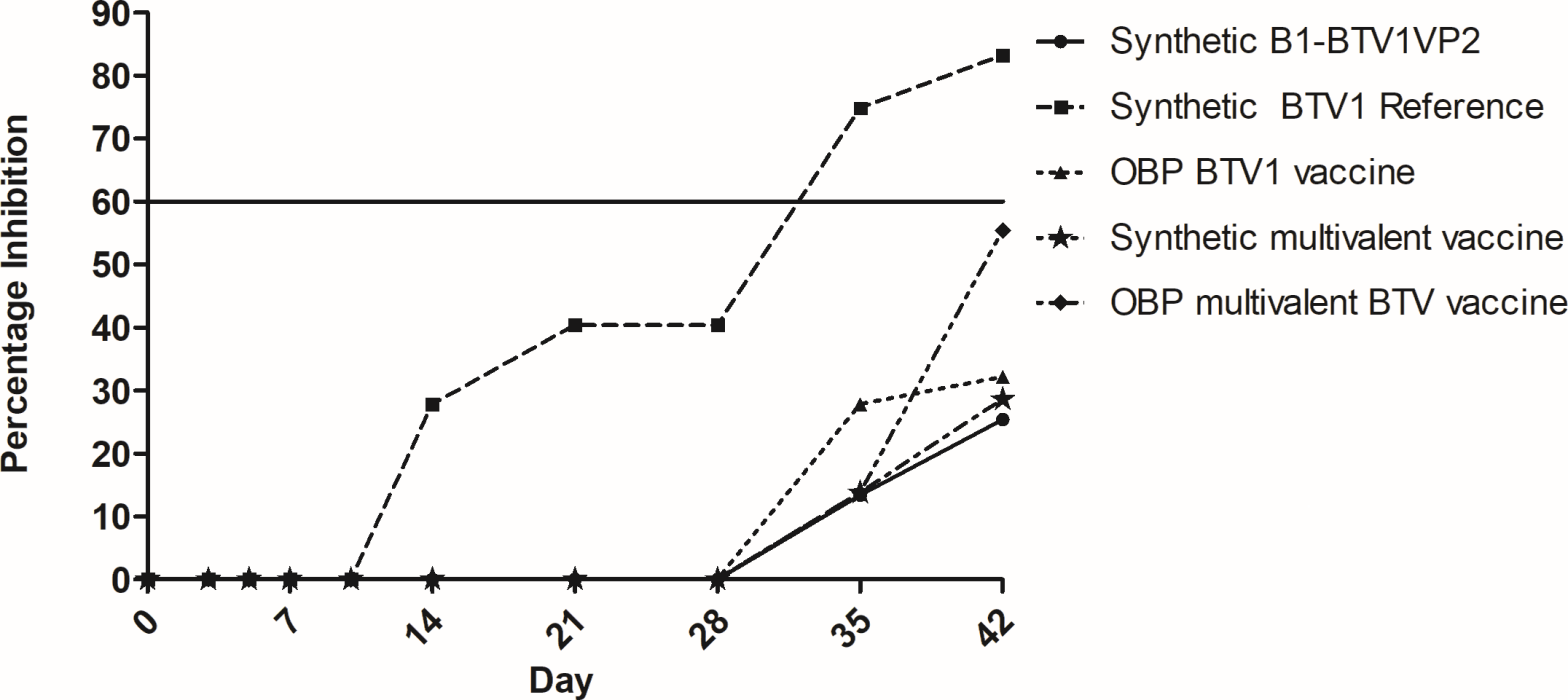
Evaluation of VP7 antibodies in vaccinated sheep. Group-specific VP7 antibodies were detected by cELISA. The threshold was 60% as specified by the BTV c-ELISA kit (VMRD, USA).

The development of group-specific VP7 antibodies was assessed using cELISA (VMRD, USA). Inhibition percentages of ≥60% confirmed seroconversion. The data in this study indicated that all sheep in the five groups were sero-negative up to day 10. An assessment of sheep inoculated with the reference strain determined onset of immunity at day 14 in two sheep, giving a group average inhibition percentage of 27.8%. Following the seroconversion of all sheep, the antibody response increased to a maximum inhibition percentage of 83.2% on day 42 (Fig. 7). In comparison, seroconversion of one sheep in the serotyped monovalent BTV1 group was detected on day 35, giving a group mean inhibition percentage of 13.4%. A peak inhibition percentage of 25.4% was calculated for the group on day 42 after a second sheep seroconverted. Similarly, on day 35, two sheep seroconverted in the licensed monovalent vaccine group with a mean value of 27.8%. A maximum value of 32.2% was determined for the two sheep on day 42. An assessment of the multivalent vaccinated groups showed that a single sheep in each group seroconverted on day 35, giving identical inhibition values of 13.8%. On day 42, two sheep that seroconverted in the multivalent synthetic vaccine group gave a mean value of 28.6%. In comparison, four sheep in the multivalent positive control group gave a peak inhibition value of 55.4%. In summary, two sheep each in the synthetic monovalent, multivalent vaccine, and monovalent licensed groups seroconverted by day 42 in comparison to four sheep in the multivalent positive control group. With the exception of the synthetic reference strain group, all calculated group inhibition percentages were below the inhibition percentage cut-off of 60%.

**Fig 8:**
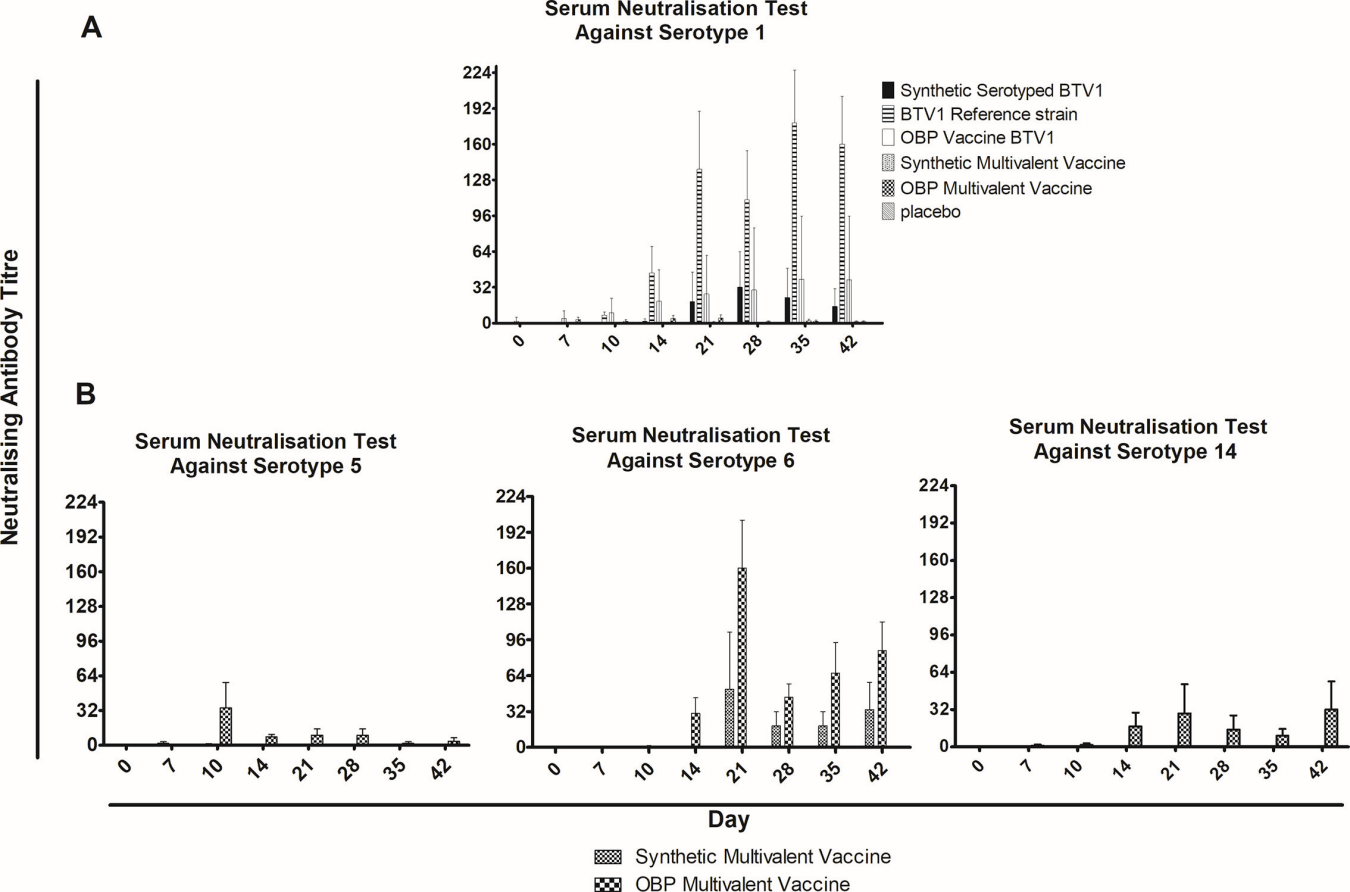
Kinetics of BTV serotype 1, 5, 6, and 14 neutralizing antibodies. Sheep were vaccinated subcutaneously with synthetic or licensed vaccines at 1.00E + 04 PFU/dose. Sera were collected on the days indicated and evaluated for neutralizing antibodies against serotypes 1, 5, 6, and 14. The reciprocal of the highest serum dilution that gave 50% protection against the virus was determined as the antibody titer. The mean serum titer was determined for each day assessed. The error bars indicate the standard deviation.

Serotype-specific nAbs induced by VP2 protein (7) were examined in vaccinated sheep. A serum antibody titer of 4 denoted seroconversion. The onset of nAbs in sheep vaccinated with the _B1_BTV1VP2 gave an average titer of 1.6 on day 14 (Fig. 8A). The nAb response peaked on day 28 with a titer of 32 before decreasing to 14.8 on day 42. In the corresponding monovalent licensed vaccine group, the maximum average nAb titer of 39.2 determined on day 35 decreased slightly to 38.8 on day 42. In comparison, sheep inoculated with the synthetic reference strain had a peak average nAb titer of 179.2 on day 35, which declined slightly on day 42 to 160. In the multivalent synthetic and licensed vaccine groups, onset of immunity against BTV1 was on days 21 and 7, respectively, giving titers of 0.8 and 3.2. A final average titer of 1.2 was determined in both multivalent vaccine groups.

The development of nAbs against serotypes 1, 5, 6, and 14 following multivalent vaccination was examined. Sheep vaccinated with the synthetic multivalent vaccine developed nAbs against BTV6 on day 21, with an average titer of 52 that decreased to 33.6 on day 42 (Fig. 8). In comparison, sheep in the positive multivalent control group seroconverted on day 10 with low titers of 0.8. The nAb titer peaked at 160 on day 21, then decreased to a final titer of 86.4. Neutralization antibody development against serotypes 5 and 14 was not induced by the synthetic multivalent vaccine. In contrast, the licensed multivalent vaccine induced serotype 5 and 14 nAbs development on days 10 and 7 with average titers of 34.4 and 1.2, respectively. The average titers were determined at 3.6 and 32, respectively, on day 42.

Additionally, cross-neutralization against serotypes 2, 3, 8, 9, 10, and 13 was evaluated in day 28 sera following vaccination with multivalent vaccines. Cross-neutralization was demonstrated only against serotype 10 following vaccination with the licensed multivalent vaccine. An average titer of 32.8 was determined. Cross-neutralization was not observed following vaccination with the synthetic vaccine against all serotypes evaluated.

## DISCUSSION

The first plasmid-based reverse genetics platform for the rescue of dsRNA viruses from cloned cDNA was developed for mammalian reoviruses (genus *Orthoreovirus*) (49). Since then, the technology has been implemented for the recovery of other viruses in vaccine development. A plasmid DNA-based reverse-genetics system for the rescue of orbivirus was developed in 2015 (37). Reverse-genetics platforms allow the introduction of desired characteristics for enhanced vaccine performance while maintaining the replication capacity of the viruses. Rescued dsRNA viruses with specific mutations have been successful for viruses, such as Tarumizu tick virus (genus *Coltivirus*) (50), mammalian orthoreoviruses (genus *Orthoreovirus*) (49), and rotaviruses (genus *Rotaviru*s) (51). Here, we evaluated the plasmid DNA-based reverse genetics platform for orbiviruses (37) as an alternative strategy to manufacturing live-attenuated bluetongue vaccine. Application of this strategy for synthetic BT vaccine manufacturing was aimed at allowing a rapid production process with a common virus backbone. Synthetic antigens were rescued by exchanging outer capsid proteins (VP2 and VP5) of different serotypes on the BTV1 backbone. The following serotyped viruses were created: _B1_BTV1VP2 (OBP vaccine strain), _B1_BTV5VP2, and _B1_BTV14VP2. Recovery of _B1_BTV6VP2VP5 was successful by exchanging segments 2 and 6 on the backbone. Serotypes 3, 4, 7, and 12, however, could not be rescued by exchanging one or both outer capsid proteins following several attempts. The unsuccessful rescue of serotyped synthetic BTV7 and BTV12 (South African reference strains) was previously reported by Nunes et al. in 2014 (29). It was speculated that the introduction of segments 2 and 6 to the backbone of a different serotype that evolved independently possibly contributed to their incompatibility during rescue, cell attachment, entry, or assembly of the new virions (52, 53). The results suggested that antigens have serotype-specific structural requirements for stable assembly. Feenstra et al. (2015) could not rescue serotypes 16 and 25 by exchanging VP2 on a BTV1 backbone. However, BTV25 was rescued by exchanging both capsid proteins. The rescue of a synthetic antigen expressing a chimeric BTV1/16 VP2 protein with serotype 1 and 16 neutralizing epitopes was achieved (54). Although BTV4 serotyped antigen could not be rescued in this study, previous researchers demonstrated the feasibility of serotyped BTV4 rescue on a BTV1 backbone using an RNA-based reverse genetics platform (29, 31, 54). The sequence variation between selected segment 2 of the OBP vaccine strain in this study and the South African reference strain used in previous studies (29, 54) may have contributed to the unsuccessful rescue. In South Africa, where BT is endemic, the synthetic vaccine must be protective against all 22 circulating serotypes. Further optimization is required to allow rescue of all other serotypes for vaccine development using this platform.

A TEM evaluation of the synthetic and commercial vaccine virus particles gave broad size ranges that were comparable. An assessment of the plaque morphology of both vaccine types indicated that registered vaccine strains, with the exception of BTV14, formed larger plaques in comparison to synthetic antigens (Table 4). Erasmus (1973) reported that African horse sickness virus (AHSV) variants with larger plaque sizes did not induce clinical disease in contrast to variants with smaller plaques (55). This observation was corroborated in subsequent AHSV studies (56). Similar observations were reported by Howel and Verwoerd when egg-adapted BTV strains with a high passage history formed larger plaques than tissue culture-adapted strains with a low passage history (57). Likewise, Janowicz and colleagues demonstrated that a pathogenic BTV8 strain with a low passage history had small plaques. When passaged multiple times, the strain was attenuated and produced larger plaques. By substituting segment 2 of the highly passaged strain onto the backbone of the low passage pathogenic strain, the plaque size of the recombinant antigen was large, and no fatalities were observed in IFNAR^−^/^−^ mice (58). Although smaller plaque sizes may be interpreted as being indicative of pathogenicity or virulence, in this study, the backbone serotype was a reference strain that has been partially attenuated through multiple passages. The implications of the reduced plaque sizes of the serotyped antigens were evaluated in safety studies in the target host.

The performance of synthetic viruses was compared to that of the registered vaccine strains using real-time cell analysis, as well as growth curves generated on Vero cells in 96-well plates, in order to evaluate the robustness of this platform in a vaccine-manufacturing environment. The synthetic BTV1 (with segment 2 from the OBP vaccine strain) and reference strain had a more rapid growth rate, while the rates of synthetic serotypes 5 and 6 were slower. Serotype 14 synthetic and vaccine strains had indistinguishable growth rates. An evaluation of yields at 72 h showed that serotype 5 vaccine types were comparable, while synthetic BTV14 had higher titers than the vaccine strain. Conversely, the yields of synthetic serotypes 1 (2.15E + 04 TCID_50_/mL) and 6 (3.78E + 02 TCID_50_/mL) were lower than those of the vaccine strains (BTV1 vaccine 6.58E + 05 TCID_50_/mL; BTV6 vaccine 1.70E + 05 TCID_50_/mL). The primary focus on improved vaccine platforms has always been on safety and maintaining efficacy that is comparable to LAVs. Implementation of newly developed vaccine platforms is often excluded on the basis of the production cost, particularly for veterinary medicines. In order for the reverse-genetics platform to be considered in a pharmaceutical environment, yields must be comparable to the currently established method to avoid production losses. In this study, the serotypes with lower yields will require further optimization to ensure titers are similar to those generated on the current platform used for the production of vaccines.

The shelf life of both vaccine types was investigated under three temperatures (4, −40, and −80°C) and three different conditions (unformulated, formulated with a stabilizer, and formulated and lyophilized). We established that traditional vaccines maintained higher titers across the storage conditions examined. Following storage, differences of up to four logs were observed when compared to synthetic vaccines. This correlated with the higher quantities of disassembled synthetic virus particles observed using TEM over a 4-week period at the three temperatures evaluated. The shelf life of unformulated antigen could potentially be extended by storage at the cold chain temperature of 4°C. We further showed that 4 and −80°C were optimum storage temperatures for antigens formulated with a stabilizer. In the pharmaceutical industry, stability may be enhanced by lyophilization (59). The data presented here indicated lyophilized antigens maintained the titer in the 10 week period of assessment with the exception of synthetic BTV1.

The safety and immunogenicity of synthetic monovalent and multivalent vaccines were investigated in the target host. The monovalent and multivalent synthetic vaccines were determined to be safe in sheep, as they did not induce temperature reactions, viremia, or BTV clinical signs. The licensed monovalent and multivalent vaccines were well tolerated as demonstrated by the absence of major temperature reactions and clinical signs. This was in contrast to the viremia and temperature reactions observed in sheep inoculated with the low-attenuation synthetic BTV1 reference strain. When the immune response following vaccination was evaluated, the synthetic vaccine elicited nAbs against serotypes 1 and 6 but not 5 and 14. Previous homologous and heterologous challenge studies demonstrated protection or partial protection in sheep with low nAb titers (33, 60). Furthermore, an inactivated serotyped vaccine that did not elicit nAbs was protective in the target host following challenge (29). Cell-mediated immunity (CMI) induced by VP2 and NS1 was speculated to have protected animals against the challenge. Hence, undetectable or low nAbs titers are not always indicative of an unprotective vaccine (29, 33). Investigations of CMI induced by the candidate synthetic vaccine would be required to further assess the vaccine’s immunogenicity.

The vaccine dose administered in this study was lower than that investigated in previous studies with or recombinant vaccines (61). A higher dose may have been required to elicit nAbs against all serotypes. The assessment of _B1_BTV5VP2 *in vitro* demonstrated a less-rapid growth profile in comparison to the commercial vaccine. van Rijn et al. (2021) postulated that a candidate vaccine antigen’s growth profile *in vitro* may predict its potency *in vivo* (33). It is, therefore, possible that the _B1_BTV5VP2 vaccine exhibited a slower replication profile in sheep that negatively affected nAb development. Furthermore, since the candidate vaccine in the study was replication-competent, a single vaccine dose was administered. A single dose, however, may have been insufficient to elicit a humoral immune response. It is possible that the serotyped vaccine may not have replicated efficiently in sheep. Further research investigating the prime-boost strategy applied with replication-incompetent vaccines may be required.

In the South African commercial BTV vaccine, serotypes 1, 6, and 14 are formulated in vaccine bottle A, while serotype 5 is in bottle C. Bottle A serotypes were reported to be of lower attenuation and slower replication (62). In the candidate vaccine, the common backbone rendered the replication machinery identical, with the exception of VP2 and VP5 from the respective serotypes. Interference during replication was, hence, maintained at a minimum. It was speculated that the vaccine serotype combination may have influenced viral replication and the subsequent immune response. In this study, for instance, BTV1 nAbs titers obtained in the monovalent vaccine were higher than those observed in the multivalent formulation. It is possible that slow replication due to interference from serotypes in the vaccine combination may negatively influence the subsequent immune response in the host. This could possibly account for the absence of seroconversion against serotypes 5 and 14. A multivalent ECRA vaccine with serotypes 1, 2, 4, 10, 13 and 21, however, was successfully protective against challenge with serotypes 2, 4, and 8 (31). Recently, an ECRA vaccine formulated with serotypes 1, 2, 3, 4, and 8 protected against the challenge with serotypes 2 and 8 (33). More work may be required with regard to vaccine combinations.

In conclusion, this study has demonstrated that reverse genetics can be utilized as a platform for the manufacturing of synthetic vaccines that are safe and immunogenic. Application of this platform is ideal for areas where a small number of serotypes are required in the vaccine formulation. Use of the platform in endemic areas, such as South Africa, with a high number of circulating serotypes requires that the stereochemistry of serotypes that could not be assembled be investigated. Furthermore, production yields and product shelf life require optimization for use in commercial manufacturing of BTV vaccine.

## Data Availability

Data generated are available in the article and associated supplemental tables and figures.

## SUPPLEMENTAL MATERIAL

Supplemental material jvi.00139-25-s0001.docx Table S1; Figures S1 to S3.

ASM does not own the copyrights to Supplemental Material that may be linked to, or accessed through, an article. The authors have granted ASM a non-exclusive, world-wide license to publish the Supplemental Material files. Please contact the corresponding author directly for reuse.
