# Supplementary material for: Plasmid DNA-based reverse genetics as a platform for manufacturing of bluetongue vaccine
Source: J Virol. 2025 Mar 6;99(4):e00139-25. doi: 10.1128/jvi.00139-25 (PMC11998535; doi:10.1128/jvi.00139-25)
Supplement: Supplemental material — Table S1; Figures S1 to S3. [file jvi.00139-25-s0001.docx]

**Plasmid DNA-based reverse genetics as a platform for manufacturing of Bluetongue Vaccine: Supplementary Tables and Figures**

**Table S1** Animal Welfare Monitoring Sheet for BTV Disease

| **Numerical Score** | **Nose** | **Mouth** | **Hooves** |
| --- | --- | --- | --- |
| 0 | Normal | Normal | Normal |
| 1 | Slight hyperaemia | Slight hyperaemia | Slight hyperaemia of the coronary band. |
| 2 | Hyperaemia & slight erosion | Slight hyperaemia & erosions at mucocutaneous junction of upper lip and swelling of mouth | Slight hyperaemia & erosions at mucocutaneous junction of upper lip and swelling of mouth |
| 3 | Erosion & petechiae | Hyperaemia, erosion and slight cyanosis of the mucosa | Hyperaemia of the coronary band with petechiae |
| 4 | Severe erosion, hyperaemia & haemorrhages | Severe erosion and cyanosis | Severe hyperaemia of the coronary band with petechiae (might become streaky) |

**Plasmid DNA-based reverse genetics as a platform for manufacturing of Bluetongue Vaccine: Supplementary Figures**

**
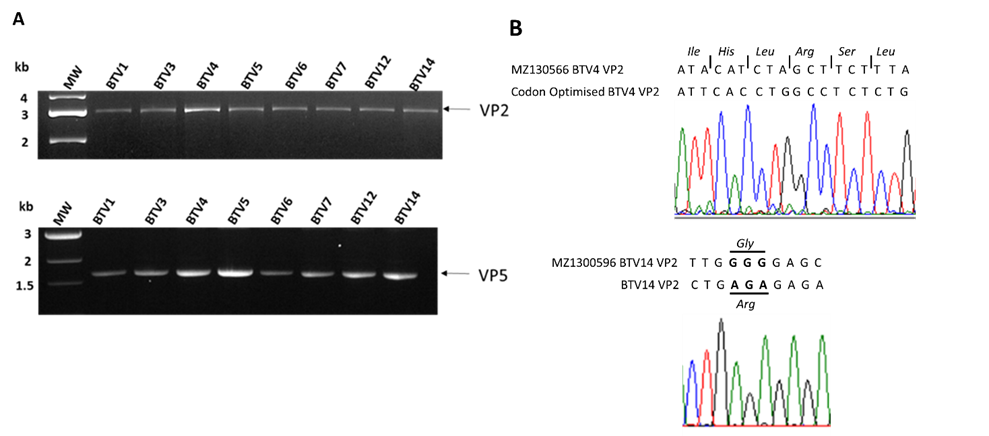
**

Figure S1. Construction of reverse genetics plasmids for recovery of synthetic viruses. (A) Reverse genetics plasmids expressing the BTV outer capsid proteins were PCR-amplified from cDNA or optimised synthetic genes. Gel-purified amplicons were separated by electrophoresis on ethidium bromide-stained 1% (w/v) agarose gels. MW represents the DNA 1Kb marker (New England Biolabs) used. (B) Amino acid sequence confirmation of the constructs gave the expected sequences for the codon-optimised segment 2 constructs. The chromatogram of BTV4 VP2 of the codon-optimised sequence is shown. A single non-synonymous mutation was identified on segment 2 of BTV14. The amino acid of the parental vaccine and construct sequence is shown in italics. Constructs with expected sequences were selected for virus rescue.


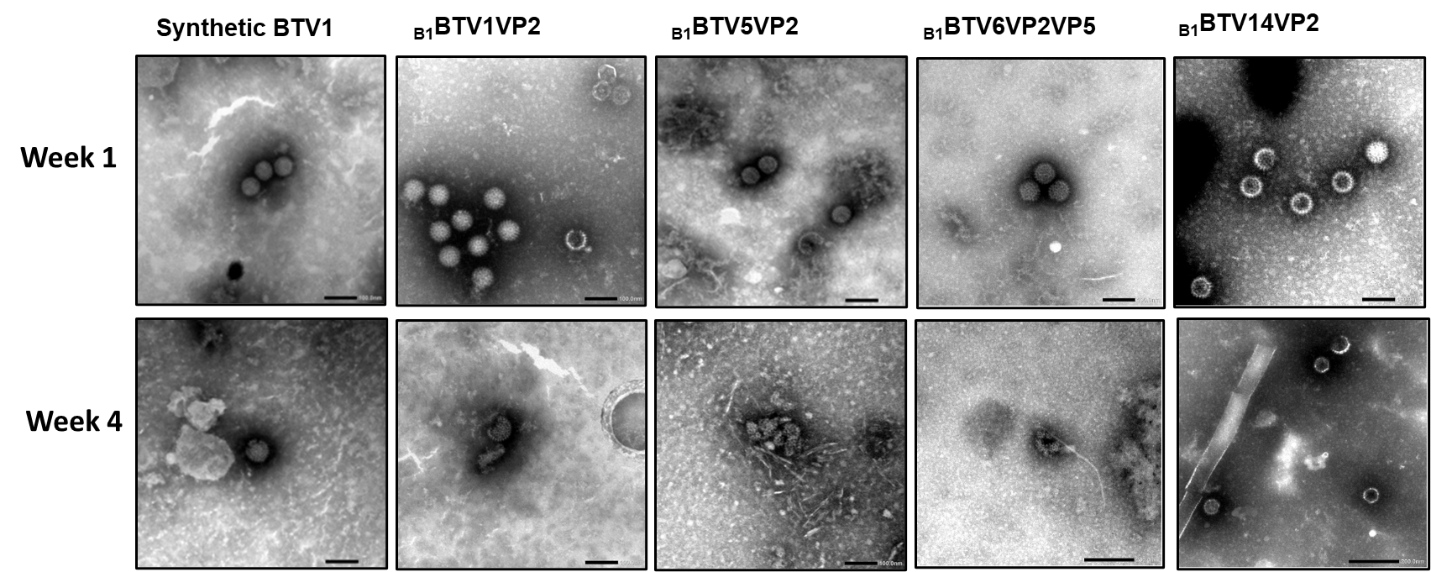


Figure S2. Stability assessment of synthetic viral particles using transmission electron microscopy. Viral particles were viewed weekly for four weeks. Fully assembled viruses were observed in high concentrations in week 1. Particles at varying degrees of disassembly were visualised in increasing quantities in the fourth week period of analysis.

Figure S3. Average clinical scores of vaccinated sheep. The average clinical score of sheep vaccinated with synthetic or licensed BTV1 monovalent or multivalent vaccine was calculated following an assessment of the body temperature, nose, mouth and hooves. The clinical scores observed 21 days following vaccination are depicted. The error bars indicate the standard deviation.
